# Post-error slowing in sequential action: an aging study

**DOI:** 10.3389/fpsyg.2014.00119

**Published:** 2014-02-18

**Authors:** Marit F. L. Ruitenberg, Elger L. Abrahamse, Elian De Kleine, Willem B. Verwey

**Affiliations:** ^1^Department of Cognitive Psychology and Ergonomics, University of TwenteEnschede, Netherlands; ^2^Department of Experimental Psychology, University of GhentGhent, Belgium; ^3^Department of Psychology, Health and Technology, University of TwenteEnschede, Netherlands

**Keywords:** post-error slowing, cognitive control, aging, motor skill, sequencing learning

## Abstract

Previous studies demonstrated significant differences in the learning and performance of discrete movement sequences across the lifespan: Young adults (18–28 years) showed more indications for the development of (implicit) motor chunks and explicit sequence knowledge than middle-aged (55–62 years; Verwey et al., 2011) and elderly participants (75–88 years; Verwey, 2010). Still, even in the absence of indications for motor chunks, the middle-aged and elderly participants showed some performance improvement too. This was attributed to a sequence learning mechanism in which individual reactions are primed by implicit sequential knowledge. The present work further examined sequential movement skill across these age groups. We explored the consequences of making an error on the execution of a subsequent sequence, and investigated whether this is modulated by aging. To that end, we re-analyzed the data from our previous studies. Results demonstrate that sequencing performance is slowed after an error has been made in the previous sequence. Importantly, for young adults and middle-aged participants the observed slowing was also accompanied by increased accuracy after an error. We suggest that slowing in these age groups involves both functional and non-functional components, while slowing in elderly participants is non-functional. Moreover, using action sequences (instead of single key-presses) may allow to better track the effects on performance of making an error.

## Introduction

The phenomenon of post-error slowing refers to the tendency of participants to slow down on the current trial after having committed an error on the previous trial (Rabbitt and Rodgers, 1977; Notebaert et al., 2009; e.g., Danielmeier and Ullsperger, 2011; Dutilh et al., 2012a, 2013; Houtman et al., 2012; Houtman and Notebaert, 2013). Ample studies have focused on post-error slowing in relatively simple reaction time (RT) tasks (e.g., single key press), but to our best knowledge this phenomenon has never been examined for more complex sequencing skill—even though it seems plausible that slowing may also be observed at the sequential level. We will refer to this notion as post-error *sequence* slowing. The present study examines for the first time the consequences of making an error on the performance of relatively complex movement sequences. To this end, we (a) examine whether post-error slowing also occurs at the sequence level, and (b) investigate whether or not such slowing is functional (i.e., attributable to strategically increased control). A further aim of the study is (c) to investigate whether age-related changes occur in post-error sequence slowing, as previous studies have shown that aging affects post-error adaptations in simple RT tasks (e.g., Band and Kok, 2000; Gupta et al., 2009; Dutilh et al., 2013) as well as sequencing skill (Voelcker-Rehage, 2008; e.g., Verwey, 2010; Verwey et al., 2011). Below, we will first elaborate on theories on post-error slowing, and we will then describe our previous work on sequence learning and aging.

### Post-error slowing

Several explanations for the emergence of post-error slowing in simple RT tasks have been put forward. Here, we follow the classification of Houtman and Notebaert (2013) and differentiate between functional and non-functional accounts for post-error slowing. Functional accounts postulate that error processing and related adjustments are intended to improve performance on subsequent trials. Following an error, participants strategically put more emphasis on response accuracy to prevent future errors (i.e., post-error accuracy increase at the cost of a decrease in RT). The most commonly accepted functional explanation for post-error slowing is the *control hypothesis* which is based on the conflict monitoring theory (Botvinick et al., 2001). It postulates that people continuously monitor their performance and that control levels are flexibly adjusted to environmental demands in order to optimize performance. Specifically, making an error results in the concurrent activation of both the correct and an incorrect response (i.e., response conflict), and the detection of this response conflict leads to increased response thresholds—and thus to more accurate yet slower performance to reduce the likelihood of committing another error (cf. speed-accuracy trade-off).

Non-functional accounts for post-error slowing, in contrast, explain post-error slowing in terms of reduced cognitive processing after errors. These non-functional accounts predict post-error slowing as well as a post-error accuracy decrease. According to the *orienting account* (Notebaert et al., 2009), an error is an infrequent event which automatically captures attention (i.e., an orienting response) and thus distracts attention away from the task itself. In line with this notion, Notebaert et al. (2009) demonstrated that when more erroneous than correct responses are given—so that each correct response constitutes an infrequent event—slowing followed the correct instead of erroneous responses. This suggests that it is not the error *per se* that causes the slowing, but rather the attentional orientation toward that event. A second non-functional account is the *bottleneck error-monitoring account* (Jentzsch and Dudschig, 2009). It postulates that error processing requires time and resources from a limited central capacity. This processing interferes with performance of the next trial, as fewer resources are available. Finally, according to the *malfunctioning account* reduced processing mechanisms delay the start of next trial. This could be due to persistence of the processing problem that led to an error on the previous trial (Gehring et al., 1993; Gehring and Knight, 2000) or overcoming the disappointment of making an error (Rabbitt and Rodgers, 1977).

It is important to note that these functional and non-functional accounts are not necessarily mutually exclusive. Previous studies on post-error slowing in simple RT tasks suggested that participants may first experience an orienting response following an error, and then later—if time allows it—strategically adjust their performance (Jentzsch and Dudschig, 2009; cf. Danielmeier and Ullsperger, 2011; Houtman and Notebaert, 2013). In fact, this may especially hold true for post-error slowing in familiar movement sequences, as possibly the longer lasting overall response time for these sequences may allow for more opportunity to benefit from strategically implemented control. The present study contributes to the existing literature on post-error slowing by testing the aforementioned explanations of post-error slowing with respect to sequential action across the lifespan. We now first outline previous work in this domain.

### Sequential skill across the lifespan

A paradigm for assessing the learning and performance of movement sequences is the discrete sequence production (DSP) task, in which participants practice the execution of one or more series of 3–6 key presses. Initially, responses are signaled by key-specific stimuli, but with practice the sequence(s) can be increasingly performed without heavy reliance on these stimuli (beyond the first one). The performance level where execution has become highly automatized and is no longer stimulus-based, may be referred to as sequence skill. It has often been recognized that task performance—even at the level of skill—is not typically process-pure: it involves both implicit/automatic and explicit/controlled processes (Jacoby, 1991; e.g., Destrebecqz and Cleeremans, 2001). To acknowledge this the *dual processor model* of sequence skill (Verwey, 2001; for a recent review see Abrahamse et al., 2013) postulates that sequencing performance results from constant interactions between a cognitive processor and a motor system. The role of the cognitive processor differs between early and late practice phases. Initially, it is responsible for the translation from a stimulus to the appropriate response; it selects the to-be-executed response, and then prompts the motor system to execute it. In this phase, movement sequences are said to be performed in the reaction mode. With more practice, motor chunks develop that allow fixed series of key presses to be selected and loaded by the cognitive processor into a motor buffer as if they constitute a single response. The motor system then reads the information from the motor buffer and executes the series in a relatively automatic fashion (i.e., the chunking mode). During such execution by the motor system, the cognitive processor can still engage in online S-R translations to assist the motor system. This leads to a race between response selection by the cognitive processor and response triggering by the motor system, resulting in the fastest possible responses (i.e., statistical facilitation; Verwey, 2001).

In all, one may define sequence skill in the DSP task as a complex mixture of implicit/automatic and explicit/controlled processes. The implicit/automatic processes include execution by the motor system, and possibly associative learning at the level of the cognitive processor both between subsequent stimulus-response events (see Abrahamse et al., 2010; Verwey and Abrahamse, 2012) and between successive motor chunks (Verwey et al., 2011, 2014). The explicit processes include sequence selection and online S-R translations (especially early on in practice, because this process may automatize over time; Verwey et al., 2011) by the cognitive processor (Abrahamse et al., 2013). In the present study we focus on post-error behavioral adjustments, which may also be assigned to the cognitive processor. As post-error slowing has not been explored in studies on discrete sequence skill before, it may give us more insight in the mechanisms underlying sequential movements.

Previous studies showed differences in the learning and performance of discrete movement sequences across the lifespan: young adults (18–28 years) showed more indications for the development of (implicit) motor chunks and explicit sequence knowledge than middle-aged (55–62 years; Verwey et al., 2011) and especially elderly participants (75–88 years; Verwey, 2010). Still, the middle-aged and elderly participants showed performance improvements which were attributed to another (implicit) sequence learning mechanism in which individual responses are primed by implicit sequential knowledge (i.e., the associative mode).

In addition to these age-related differences in sequencing performance, age-related differences have also been demonstrated for post-error slowing in simple RT tasks, in the sense that post-error slowing has been found to be larger for older than younger adults (Smith and Brewer, 1995; Gehring and Knight, 2000; e.g., Dutilh et al., 2013). It has been suggested that young adults try to balance speed and accuracy to realize optimal performance, so that performance slows a little after making an error, while older adults tend to emphasize accuracy over speed and become even more cautious after making an error (e.g., Smith and Brewer, 1995; Starns and Ratcliff, 2010). As aging thus seems to modulate both the relative contributions of implicit and explicit mechanisms to sequential action—as put forward in the dual processor model—and the magnitude of post-error slowing at the simple RT level, it seems plausible that post-error slowing in sequencing performance will also vary among different age groups.

### The present study

In the present study we aimed, first, to demonstrate post-error slowing for relatively complex response sequences—as opposed to slowing of single responses in simple RT tasks. We examined the effect of making an error on mean RTs during sequencing performance. It was hypothesized that sequences are performed slower when an error is made on the preceding sequence as compared to when the preceding sequence is performed correctly. If so, we were further interested to see whether such slowing would be limited to the first key press of a sequence, or whether it would endure across multiple key presses. According to the DPM, making an error at least slows the first key press of a following sequence, as error-processing hinders sequence selection by the cognitive processor. As the cognitive processor is also involved in direct S-R translations to determine subsequent responses (reaction mode and associative mode; cf. elderly participants) and in online S-R translations (chunking mode; young adults and to a lesser extent middle-aged participants), other key presses within the sequence are expected to be slowed as well. Second, we explored whether aging affects post-error sequence slowing, and hypothesized that the magnitude of slowing would increase with age. We investigated post-error changes in accuracy for each age group to discriminate between functional sequence slowing (predicting a post-error accuracy increase) and non-functional sequence slowing (predicting a post-error accuracy decrease). In the discussion, we interpret the results both in terms of (non-) functional post-error adjustments and within the existing framework of the dual processor model.

## Methods

### Participants

The experimental data of 24 young adults (mean age = 22, range = 18–28, 16 women), 24 middle-aged participants (mean age = 58, range = 55–62, 10 women) and 24 elderly participants (mean age = 79, range = 75–88, 13 women) that had been collected in the practice phase of the Verwey (2010) and Verwey et al. (2011) studies were used for the analyses.

### Task and procedure

The task and experimental procedure are described in greater detail in the studies of Verwey and colleagues. Here we provide the most important information regarding the experiment. Participants in were instructed to place their left and right ring, middle, and index fingers on the d, f, g, j, k, and l keys of a notebook computer keyboard. Six black horizontally aligned square stimulus placeholders were displayed against a white background. Between the third and fourth placeholder a small gap appeared with the letter “H” in the middle so that it mimicked the keyboard lay-out. When one of the placeholders was filled with green, participants responded to the stimulus by depressing the spatially corresponding key (e.g., d for the leftmost square). Directly after the correct key had been pressed, the next stimulus in the sequence was presented by filling another placeholder with green.

Each participant was presented one sequence of three stimuli and one sequence of six stimuli. Correctly pressing the corresponding keys thus resulted in a fixed sequence of three key presses and a fixed sequence of six key presses. For half of the participants in each age group, the 6-key sequence contained a pause between the response to the third stimulus and the presentation of the fourth stimulus (i.e., the prestructured group) to impose a segmentation structure onto the sequence (e.g., Verwey, 1996). For the other half of the participants the 6-key sequence did not include a pause and the next stimulus of a sequence was thus presented as soon as the correct key was pressed (i.e., the unstructured group). Across all participants, the key presses (and thus fingers) in the sequences were counterbalanced across sequential positions to avoid finger-specific effects on response times. For example, one participant practiced the sequences KFGDJL and FKL, the next participant practiced LGJFKD and GLD, and so on. Each of the two sequences that a participant practiced started with a different key press, so that the to-be-performed sequence could be selected on basis of the first stimulus. Participants practiced their sequences during six blocks that each included the presentation (in random order) of 24 3-key sequences and 24 6-key sequences. In total, participants thus practiced each of their sequences 144 times. At the end of each block participants were presented their mean reaction time and error percentage.

Before presentation of the first stimulus of a sequence, the six empty placeholders were displayed for 1000 ms. Directly after a participant pressed the correct key, the next stimulus of the sequence appeared. Following each correctly executed sequence the display was erased white for 2000 ms to indicate completion of the sequence. Pressing an incorrect key resulted in an error message for 500 ms. The ongoing sequence was then terminated and followed by the presentation of the next sequence started.

### Data analysis

We first calculated mean response times (RTs) per key press for the 3-key and 6-key sequences for every participant in each block. RT was defined as the time between stimulus presentation and depression of the correct response key. Sequences in which one or more errors had been made were omitted from the RT analyses. In addition, sequences were omitted from the RT analyses when the total execution time exceeded more than 2.5 standard deviations from the mean across participants in a particular age group. This was done separately for the 3- and 6-key sequences per block and resulted in the removal of less than 1% of the sequences.

To investigate post-error slowing we calculated mean RTs per key press for sequences that were performed immediately following a sequence in which an error was made, as well mean RTs per key press for sequences that were performed immediately following another correctly performed sequence. As each age group included participants who did not make any errors in (one of) their blocks, calculating post-error trials per block would result in the total data of these participants being excluded from the overall analyses. To maximize the number of included participants, we pooled together the data of the first three blocks, and did the same for the last three blocks.

Notably, even after this procedure, the data of some participants indicated that they had not made any errors in the first or last three blocks. Consequently, the analyses below could not always be based on the data of all participants (the number of included participants for each analysis is stated in the results section). Moreover, the analyses for 3- and 6-key sequences were not always based on exactly the same group of participants. RTs of the 3-key and 6-key sequences were subjected to separate mixed factorial analyses of variance (ANOVAs) with Trial type (2; post-error vs. post-correct), Block (2; first three blocks vs. final three blocks) and Key position within the sequence (resp. 3 and 6; hereafter referred to as Key) as repeated measures and Age group (3: young adults vs. middle-aged vs. elderly) as between-subject variable. The analyses of the 6-key sequences additionally included Pause (2: pause vs. no pause) as a between-subject variable. For the 3-key sequence 90% of the cells included more than one case (i.e., post-error trial), and for the 6-key sequence 85% of the cells included more than one case. Table 1 provides an overview of the mean number of post-error trials on which the means in each age group were based, as well as the standard deviations and range of post-error trials for each sequence per age group. Below, we only report main and interaction effects of Trial type, as other main and interaction effects have been reported elsewhere (Verwey, 2010; Verwey et al., 2011).

**Table 1:** **The mean number of cases (i.e., post-error trials) per age group for the 3-key and 6-key sequences, and their standard deviations (*SD*) and range**.

		**Mean**	***SD***	**Range**
3-key sequence	Young adults	5.96	3.40	1–16
	Middle-aged	4.30	3.00	1–11
	Elderly	11.28	9.30	1–40
6-key sequence	Young adults	3.60	2.47	1–13
	Middle-aged	3.37	2.15	1–9
	Elderly	7.62	6.01	1–30

## Results

### Post-error sequence slowing

**Figure 1:**
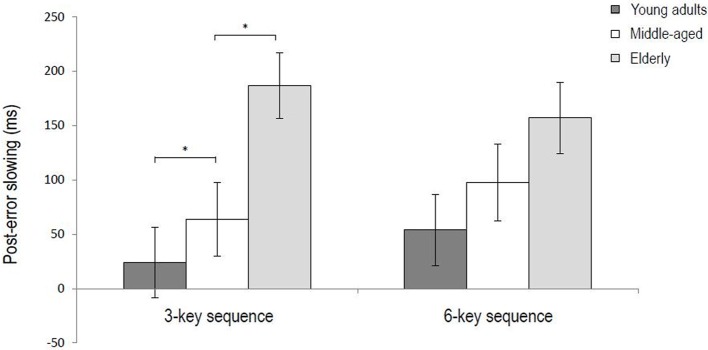
Post-error sequence slowing for the 3-key and 6-key sequences as a function of age group (^*^p < 0.05). Error bars represent standard errors.

For the 3-key sequence, the data of 21 young adults, 19 middle-aged and 24 elderly participants were included in the analysis (i.e., 4 young adults and 5 middle-aged participants were excluded). Results of the ANOVA showed that post-error sequences were generally performed slower than post-correct sequences (688 ms vs. 597 ms), *F*_(1, 61)_ = 24.21, *p* < 0.001, η^2^_*p*_ = 0.28. The average post-error sequence slowing thus amounted to 91 ms. An interaction between Trial type and Age group indicated that post-error sequence slowing differed between the three age groups, *F*_(2, 61)_ = 7.43, *p* < 0.01, η^2^_*p*_ = 0.19. Slowing was significant for all age groups, *Fs* > 14.00, *ps* < 0.01, η^2^_*p*_*s* > 0.43, but as the left panel of Figure 1 illustrates post-error sequence slowing was larger for the middle-aged and elderly participants than the young adults, *Fs* > 5.08, *ps* < 0.05, η^2^_*p*_*s* > 0.11. In addition, slowing was larger for the elderly than the middle-aged participants, *F*_(1, 41)_ = 4.99, *p* < 0.05, η^2^_*p*_ = 0.10. There was no Trial type × Key interaction (*p* = 0.10), indicating that the magnitude slowing did not differ significantly between the various key presses within the sequence.

**Figure 2:**
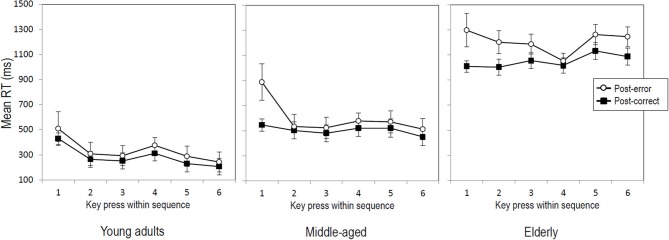
**Mean RTs per key press within post-error (open circles) and post-correct (black squares) 6-key sequences as a function of age group.** Error bars represent standard errors.

For the 6-key sequence, the data of 23 young adults, 20 middle-aged and 23 elderly participants were included. Results of the ANOVA showed that post-error sequences were generally performed slower than post-correct sequences (714 vs. 612 ms), *F*_(1, 60)_ = 28.28, *p* < 0.001, η^2^_*p*_ = 0.32. So, the average post-error sequence slowing amounted to 102 ms. An additional Trial type x Key interaction suggested that the amount of post-error slowing differed between the key presses within the sequence, *F*_(5, 300)_ = 4.00, *p* < 0.05, η^2^_*p*_ = 0.06. Further analyses showed that the interaction was no longer significant after removing the first key press of the sequence from the analysis (*p* = 0.37), while the main effect of Trial type still remained significant, *F*_(1, 60)_ = 23.52, *p* < 0.001, η^2^_*p*_ = 0.28. This indicates that across age groups making an error in a previous sequence slowed the first key press of the subsequent sequence more than later key presses of that sequence—which however were still significantly slowed (236 ms for key 1 vs. on average 76 ms for keys 2–6). Figure 2 illustrates that this applies to participants in each age group, despite different RT baselines and RT patterns for the three age groups. Results further showed a Trial type x Block interaction, *F*_(1, 60)_ = 6.27, *p* < 0. 05, η^2^_*p*_ = 0.09, suggesting that the magnitude of post-error slowing differed between the first and second half of the experiment. Although slowing was significant in both halves of the experiment (146 vs. 61 ms), *Fs* > 19.21, *ps* < 0.001, η^2^_*p*_*s* > 0.23, it was larger in the first half. Finally, results of the ANOVA on RTs in the 6-key sequence showed no significant interaction between Trial type and Age group (*p* = 0.089), indicating that the magnitude of post-error slowing did not differ between the three age groups (see Figure 1, right panel).

Finally, to examine whether baseline differences in RTs between the three age groups played a role in the observed effects, we calculated z-scores based on the factor Age group and performed the aforementioned analyses on these standardized RTs. Results showed that the main effect of Trial type remained significant for both the 3-key and 6-key sequences, *Fs* > 32.70, *ps* < 0.001, η^2^_*p*_*s* > 0.35, thus confirming post-error sequence slowing. However, we no longer observed the Trial type x Age group interaction in either of the sequences (*ps* > 0.26). We will elaborate on the use of standardized RT scores in the discussion section below.

### Testing theories of post-error slowing

To explore whether post-error sequence slowing in each age group was—to some extent—functional or not, we investigated post-error accuracy (in terms of the proportion of correctly performed sequences) and determined whether accuracy increased or decreased after making an error. An accuracy increase would support the idea that post-error slowing results from an error-driven increase in control, whereas an accuracy decrease would indicate that slowing is predominantly non-functional.

We used arcsine transformations to stabilize variances of the accuracy proportions, which is recommended for proportion data with binominal distributions (Winer et al., 1991)^1^. For the 3-key sequence, we ran an ANOVA on the arcsine transformed scores with Trial type (2; post-error vs. post-correct) and Block (2) as within-subject variables, and Age group (3) as between-subject variable. Results showed that the difference between post-error and post-correct sequence accuracy varied across the three age groups, *F*_(2, 61)_ = 9.29, *p* < 0.001, η^2^_*p*_ = 0.23. An additional Trial type × Block × Age group interaction suggested that post-error accuracy changes differed across the blocks for some age groups, *F*_(2, 61)_ = 4.35, *p* < 0.05, η^2^_*p*_ = 0.12. Planned comparisons showed that accuracy increased after making an error for young adults (post-error 0.98 vs. post-correct 0.96) and middle-aged participants (post-error 0.97 and post-correct 0.96), *Fs* > 16.42, *ps* < 0.01, η^2^_*p*_*s* > 0.47, with no differences between blocks (*p* = 0.57). For elderly participants accuracy only decreased after making an error in the first half of the experimental blocks (post-error 0.80 vs. post-correct 0.92), *F*_(1, 23)_ = 5.97, *p* < 0.05, η^2^_*p*_ = 0.21, but not the second half of the blocks (post-error 0.92 vs. post-correct 0.93; *p* = 0.31).

A similar ANOVA was performed on transformed accuracy proportions for the 6-key sequences, now also including the between-subject variable Pause (2). Again, results showed that post-error changes in sequence accuracy differed between the three age groups, *F*_(2, 60)_ = 8.75, *p* < 0.001, η^2^_*p*_ = 0.22. Specifically, accuracy increased after an error for young adults (post-error 0.96 vs. post-correct 0.93) and middle-aged participants (post-error 0.954 vs. post-correct 0.949), *Fs* > 8.66, *p* < 0.01, η^2^_*p*_ = 0.32, but remained unchanged for elderly (post-error 0.79 vs. post-correct 0.86; *p* = 0.52).

Finally, we determined the correlation between post-error slowing and overall accuracy on the 3-key and 6-key sequences. The orienting account would predict that a larger overall proportion of correctly performed sequences and thus more infrequent errors would be related to more slowing due to such errors. In line with this prediction, results showed positive correlations between accuracy on the 3-key sequence and post-error slowing on that sequence for young adults, *r*_(24)_ = 0.35, *p* < 0.05, and middle-aged participants, *r*_(24)_ = 0.37, *p* < 0.05 (both one-tailed). No significant correlation was observed for elderly participants (*p* = 0.91). Furthermore, no significant correlations were observed for the 6-key sequence (*ps* > 0.13).

In summary, the present results showed that performance of both the 3-key and 6-key sequence slowed down after participants made an error in the previous sequence. As hypothesized, we observed that post-error sequence slowing increased with age. Furthermore, we observed a post-error accuracy increase for young adults and middle-aged participants. There were no correlations between post-error slowing and overall accuracy.

## Discussion

This study examined error processing in sequence skill across various age groups. We demonstrated for the first time that post-error slowing can be observed for entire response sequences. Additionally, the present results showed that for the 3-key sequence such post-error sequence slowing was larger in older than younger adults (Smith and Brewer, 1995; Gehring and Knight, 2000; cf., Dutilh et al., 2013). We observed that across all age groups the first key press of a relatively long (i.e., 6-key) sequence was slowed more—but not exclusively—after committing an error than the other key presses. As outlined in the Introduction, both functional and non-functional explanations for post-error slowing have been proposed. Functional accounts state that slowed responding after an error results from performance adjustments that serve to avoid errors on subsequent trials and predict a post-error accuracy increase (e.g., Botvinick et al., 2001). Non-functional accounts state that slowing is the result of reduced cognitive processing and predict a post-error accuracy decrease (e.g., Rabbitt and Rodgers, 1977; Gehring et al., 1993; Gehring and Knight, 2000; Jentzsch and Dudschig, 2009; Notebaert et al., 2009). Results of the present study showed significant differences in post-error accuracy changes between the three age groups. This suggests that different mechanisms underlie post-error sequence slowing in the different age groups.

With respect to the young adults and middle-aged participants (who showed comparable results), the observation that accuracy increased after making an error is indicative of functional slowing (cf. control hypothesis; Botvinick et al., 2001). In contrast, the observation that in these age groups general accuracy of the 3-key sequence positively correlated with post-error slowing in that sequence—which is in line with the orienting account (Notebaert et al., 2009; cf. Houtman et al., 2012)—suggests that there may also be a non-functional component in post-error slowing. In line with earlier studies that concluded that functional and non-functional accounts are not mutually exclusive (Jentzsch and Dudschig, 2009; Danielmeier and Ullsperger, 2011; Houtman and Notebaert, 2013), we therefore suggest that both functional and non-functional mechanisms contribute to post-error sequence slowing in young adults and middle-aged participants. Specifically, in line with previous authors we propose that directly after an error an orienting response may first cause a short-lived state of (non-functional) attentional distraction, while other, more functional processes related to error prevention in the oncoming trial may only be effectuated when sufficient time is available on that specific trial. In the present experiment, the relatively long time (1500 ms) between an error and the presentation of the first stimulus of a subsequent sequence, as well as the fact that the subsequent trial consisted of multiple elements (i.e., at least 3 key presses) may have enabled the benefits of a functional system to take effect. It should be noted that the time interval between a response and the presentation of the first stimulus of the subsequent sequence was larger for erroneous than for correct responses. As earlier work has shown that the magnitude of post-error slowing depends on the response-to-stimulus interval (Jentzsch and Dudschig, 2009; e.g., Danielmeier and Ullsperger, 2011), this may have affected the here observed post-error sequence slowing effects in that presentation of the subsequent stimulus 1500 ms after the making of an error could have allowed for the functional system to kick in. Future studies should therefore examine post-error sequence slowing while using smaller time intervals between the making of an error and the presentation of the next stimulus.

Results of the elderly participants showed a decrease in post-error accuracy of the 3-key sequence in the first half of the experiment, but no further post-error accuracy changes in the second half of the experiment or in the 6-key sequence. We found no correlation between overall accuracy and post-error sequence slowing for this age group, which argues against the orienting account and thus is more in favor of the bottleneck account (Jentzsch and Dudschig, 2009) and the malfunctioning account (Rabbitt and Rodgers, 1977; Gehring et al., 1993). These findings challenge the idea that post-error sequence slowing reflects functionally increased control, as seems to be the case with young adults and middle-aged participants.

Finally, an alternative explanation may be proposed for our observation of post-error sequence slowing. As pointed out by Dutilh et al. (2012b, 2013), post-error slowing may be an artifact of performance improvements that occur over the course of an experiment. Specifically, most errors (and therewith post-error trials) usually occur during the beginning of the experiment when participants are still slow at responding, compared to post-correct trials that mostly occur later in the experiment when performance is generally faster. This may especially hold for DSP-like tasks, in which fixed response sequences are learned. To examine this possibility, we *post-hoc* examined post-error sequence slowing using the method proposed by Dutilh et al. (2012b). This method directly compares data of post-error trials with those of pre-error trials (as opposed to post-correct trials), so that the effect of possible performance improvements over time is eliminated. Results showed the same pattern of results as our aforementioned analyses^2^, indicating post-error sequence slowing for both the 3-key and 6-key sequence. In addition, for the 3-key sequence the magnitude of slowing increased with age. These results thus suggest that the here observed post-error sequence slowing is unlikely to be an artifact of performance improvements across practice.

### Post-error sequence slowing and cognitive processing

In this section we will interpret our observation of post-error slowing at the sequence level in terms of the dual processor model of sequencing skill (Verwey, 2001; Abrahamse et al., 2013). First, the current observation that across all age groups the first key press of a relatively long (i.e., 6-key) sequence was slowed more—but not exclusively—after committing an error than the other key presses, suggests that sequence selection and/or preparation by the cognitive processor are slowed more than execution of the elements within the sequence. Such slowing of the first key press may be due to hindered sequence selection after an error as limited cognitive resources are available (cf. non-functional accounts), or due to more careful preparation after an error due to increased control (cf. Botvinick et al., 2001). As selection and preparation are relatively simple in shorter sequences (Verwey, 1999), the first key press of the 3-key sequence was not slowed more after committing an error than the other key presses key presses of the sequence.

As outlined in the introduction, work with the DSP task has demonstrated that motor chunk development in young adults allows for the very rapid performance of key presses on the basis of internal representations by the motor system, supported by online S-R translations by the cognitive processor. We suggest that after making an error, young adults increase control and error-monitoring which then absorbs the resources of the cognitive processor—which consequently cannot engage in online S-R translations during post-error sequencing performance (Verwey, 2001; Verwey et al., 2010, 2014; cf. Abrahamse et al., 2013). A such, sequence execution is based solely on response triggering by the motor system and this results in slightly but systematically slower sequencing performance. Verwey et al. (2011) demonstrated that middle-aged participants, compared to young adults, make limited use of motor chunks for sequence execution. Moreover, the motor chunks are not as strongly developed as in young adults, so it may well be that after making an error, middle-aged participants do not trust to rely on their chunks, and strategically switch back to the associative mode or reaction mode to increase control. As a result, all key presses within the sequence are slowed.

For elderly participants, post-error sequence slowing seems to result from a non-functional processing problem that outlasts the time between the error and presentation of the first stimulus of the next sequence. In terms of the dual processor model, it seems that error processing required part of the cognitive processor's capacity, so that fewer resources remained available for the primary task (Verwey et al., 2014). This yielded slower S-R translations by the cognitive processor. However, the observation that all key presses of a sequence were slowed cannot be explained as such, as one would expect non-functional slowing to only be a brief, short-lasting effect. Another possibility, then, is that elderly no longer trusted to rely on implicit associations between sequence elements and only used explicit S-R translations for sequence execution. Consequently, they no longer experienced the beneficial effect of response priming on sequencing performance (cf. Verwey, 2010) so that responses across the entire sequence were slowed.

One issue for future research concerns the differences in baseline RT between participants of different age groups, as this could affect current interpretations. Specifically, the difference in post-error sequence slowing between the three age groups was not significant when standardized RTs were analyzed. However, it is currently unclear whether and how the processes that underlie the post-error slowing are related to overall age-related changes in response speed. This renders it difficult to determine whether or not baseline RT differences should be compensated for. Based on the dual processor model, we previously speculated that slowing observed for the elderly participants results from increased reliance on the cognitive processor—which has different roles in different age groups—and the absence of chunk-based performance (Verwey, 2010). If so, one could argue on the one hand that probably the overall higher baseline RTs does not directly relate to the processes underlying post-error slowing, rendering it not necessary to compensate for baseline RT differences by calculating standardized scores. On the other hand, it may also be that the overall increased reliance on the cognitive processor may interact with the cognitive processor's presumed role in error prevention. In the latter case, it may actually be preferred to interpret standardized scores. This issue needs further exploration.

The current results suggest that the effect of making an error on subsequent performance depends on the mechanism that underlies sequencing skill. When sequencing performance is based on motor chunk use in the chunking mode, as is the case with young adults and (to a lesser extent) middle-aged participants, cognitive control can be functionally increased to realize more accurate performance. In contrast, when responses are implicitly primed by previous responses in the associative mode, such as with elderly participants, slowing seems to be due to reduced cognitive resources (part of which are engaged in error-processing). The observed differences regarding the mechanism underlying post-error sequence slowing between young adults, middle-aged and elderly participants may result from age-related changes in the brain. Neuroimaging studies have suggested that error-related adjustments such as post-error slowing rely on a brain network including the frontal cortex and anterior cingulate cortex (Gehring and Knight, 2000; e.g., Botvinick et al., 2001; Ridderinkhof et al., 2004; Danielmeier and Ullsperger, 2011), both of which have been found to deteriorate with age (e.g., Nyberg et al., 2010; Mann et al., 2011).

Overall, the current study for the first time demonstrated post-error slowing at the response sequence level (as opposed to single RT level). We observed that discrete movement sequences were executed slower following an error in a preceding sequence as compared to following a correctly executed sequence. For young adults and middle-aged participants, post-error slowing seems to be primarily the result of strategically increased control to prevent future errors (i.e., functional slowing). Yet, non-functional attentional distraction resulting from an orienting response to the error may occur as well as. In contrast, our results suggest that for elderly participants making an error reduces cognitive resources on the next trial (i.e., non-functional slowing). The results of the young adults indicate that sequential action may be a fruitful paradigm for future investigations on the effects of making an error on performance, as well as effects of ageing in this domain.

### Conflict of interest statement

The authors declare that the research was conducted in the absence of any commercial or financial relationships that could be construed as a potential conflict of interest.

## Footnotes

^1^Accuracy proportions with a value of 1 were replaced by 0.9995.

^2^We performed the same ANOVAs as reported in the results section, now including Trial type (2; post-error trials vs. pre-error trials). Results of the 3-key sequence showed that post-error sequences were performed slower than pre-error sequence (677 vs. 594 ms), *F*_(1, 59)_ = 18.34, *p* < 0.001, η^2^_*p*_ = 0.24, thus indicative of post-error sequence slowing. Importantly, results again showed that the magnitude of post-error slowing differed between the age groups, *F*_(2, 59)_ = 5.45, *p* < 0.01, η^2^_*p*_ = 0.16. Slowing was significant in all age groups, *Fs* > 4.94, *ps* < 0.05, but increased with age. For the 6-key sequence, post-error sequences were also performed slower than pre-error sequences (715 vs. 595 ms), *F*_(1, 53)_ = 34.66, *p* < 0.001, η^2^_*p*_ = 0.39, but the magnitude of post-error sequence slowing did not differ between age groups (*p* = 0.19).
